# Comparison of Bilateral Rectus Sheath Block and Thoracic Epidural Analgesia for Postoperative Pain Control After Open Gastrectomy: A Randomized Controlled Trial

**DOI:** 10.3390/medicina61091695

**Published:** 2025-09-18

**Authors:** Janis Opincans, Igors Ivanovs, Aleksejs Miscuks, Janis Pavulans, Elina Zemite, Agris Rudzats, Zurabs Kecbaja, Aleksejs Kaminskis

**Affiliations:** 1 Department of Surgery, Riga East Clinical University Hospital, 1038 Riga, Latviajpavulans@gmail.com (J.P.); agris.rudzats@aslimnica.lv (A.R.); zurabs.kecbaja@aslimnica.lv (Z.K.); aleksejs.kaminskis@aslimnica.lv (A.K.); 2 Faculty of Medicine, University of Latvia, 1004 Riga, Latvia; aleksejs.miscuks@gmail.com; 3 Faculty of Medicine, Riga Stradins University, 1007 Riga, Latvia

**Keywords:** analgesia, laparotomic gastrectomy, bupivacaine infusion, thoracic epidural analgesia, postoperative recovery

## Abstract

*Background and Objectives:* Thoracic epidural analgesia (TEA) is considered the gold standard for postoperative pain control following major abdominal surgery. Bilateral rectus sheath block (RSB) is a promising alternative regional technique. This study aimed to compare the efficacy of RSB and TEA in managing early postoperative pain and enhancing recovery after open gastrectomy. *Materials and Methods:* Between October 2021 and December 2024, 70 patients scheduled for elective open gastrectomy were randomized into two groups: Group A (RSB with continuous bupivacaine infusion) and Group B (TEA with 10 mg bupivacaine plus 1 µg/mL fentanyl). Primary outcomes included opioid consumption within 72 h postoperatively and pain intensity measured using the visual analog scale (VAS). Statistical analysis was conducted using the Mann–Whitney U test, Friedman’s ANOVA with Bonferroni correction, and Chi-square or Fisher’s exact test for categorical variables. *Results:* A total of 64 patients were finally included (30 in RSB, 34 in TEA). VAS scores in the RSB group were significantly lower at 24 and 48 h postoperatively compared to baseline (*p* < 0.001). Between-group comparisons showed consistently lower pain scores in the RSB group at all measured time points. At 48 h, 94% of patients in the TEA group required rescue analgesia, compared to only 17% in the RSB group. Additionally, the RSB group had a significantly shorter postoperative hospital stay (mean 6 vs. 9 days) and demonstrated earlier return of bowel function. *Conclusions:* RSB is a safe and effective alternative to TEA for analgesia after open gastrectomy. It significantly lowers pain scores, reduces opioid and rescue medication use, shortens hospital stay, and enhances early recovery. Bilateral rectus sheath block with continuous bupivacaine infusion significantly lowers pain scores, reduces opioid and rescue medication use, shortens hospital stay, and facilitates early recovery.

## 1. Introduction

Several analgesic techniques are used to provide postoperative pain relief following major intra-abdominal surgeries. While these methods are highly effective, they are also associated with postoperative gastrointestinal dysfunction (POGD), commonly referred to as postoperative ileus (POI). POI is a frequent complication after major intra-abdominal procedures, with an incidence ranging from 10% to 30% [1,2,3,4]. POGD is characterized by a transient disruption in bowel motility, leading to symptoms such as nausea, vomiting, abdominal tenderness and distension, absence of bowel sounds, and delayed passage of stools [3,4]. This not only impairs patient recovery but also imposes a significant financial burden on the healthcare system due to additional laboratory investigations, radiological examinations, medication prescriptions, and prolonged hospital stays [1].

To address these challenges, multimodal analgesia has been proposed as a superior alternative to traditional pain management strategies. This approach involves the use of multiple analgesic techniques in tandem, such as local or regional anesthesia alongside medications like nonsteroidal anti-inflammatory drugs (NSAIDs) or opioids [1]. Multimodal analgesia not only improves pain control but also reduces overall opioid consumption, thereby lowering the risk of addiction and related side effects. Consequently, such an approach promotes faster recovery, earlier mobilization, greater patient satisfaction, and shorter hospital stays [1,2,5]. Thoracic epidural analgesia (TEA) has been widely regarded as the gold standard for postoperative pain management following major abdominal surgery [2]. Studies have shown that TEA provides superior pain control, enhances quality of life after laparotomy, and reduces adverse events compared to high-dose systemic opioid administration [6,7]. Nonetheless, TEA also carries potential risks. Serious complications, though infrequent, have been reported including nerve injury, epidural hematoma, and epidural abscess. More commonly, the technique has been associated with side effects such as hypotension, motor blockade of the lower limbs, and urinary retention, which regularly necessitate catheterization [2,7].

On the other hand, rectus sheath block (RSB) is an emerging and effective regional anesthetic technique that provides somatic analgesia from the xiphoid process to the symphysis pubis. It is particularly beneficial for open laparotomies, including when indicated for open gastrectomies, and serves as a promising alternative for regional anesthesia. The technique involves injecting a local anesthetic into the space between the rectus abdominis muscle and the posterior rectus sheath. This can be administered either as intermittent boluses or through continuous infusion via a catheter placed in this space during the early postoperative period [2,7,8]. RSB with continuous local anesthetic infusion still is not widely considered an alternative for pain management after laparotomies, as the method remains unpopular. Consequently, out of habit, both surgeons and anesthesiologists tend to doubt the effectiveness of local anesthesia and prefer epidural anesthesia. Therefore, the present study aimed to evaluate whether RSB administered via surgically placed catheters could serve as a safe and effective alternative to TEA for managing early postoperative pain in patients undergoing elective abdominal surgery through a midline incision. This single center randomized clinical trial focused specifically on the added benefit of bilateral RSB in aiding postoperative recovery in patients undergoing elective open gastrectomy. A distinctive feature of our approach is the use of a patented catheter insertion technique, performed directly by the surgeon during the procedure. This technique enables continuous infusion of bupivacaine, providing sustained postoperative analgesic effects.

## 2. Materials and Methods

Between October 2021 and December 2024, we planned and undertook a randomized controlled trial at the Riga East Clinical University Hospital (RAKUS) in Riga, Latvia. The study protocol was reviewed and approved by the Research Ethics Committee of the University of Latvia (decision no. 25012021, dated 25 January 2021). In accordance with the Helsinki Declaration, 2013, all participants provided written in-formed consent before taking part in the study. The trial protocol was also registered in the ClinicalTrials.gov database (ID: NCT05592496). The randomized clinical study was conducted with the approval of the Riga East University Hospital’s scientific department. The approval number is AP-21/21.

### 2.1. Patient Enrolment

**Figure 1:**
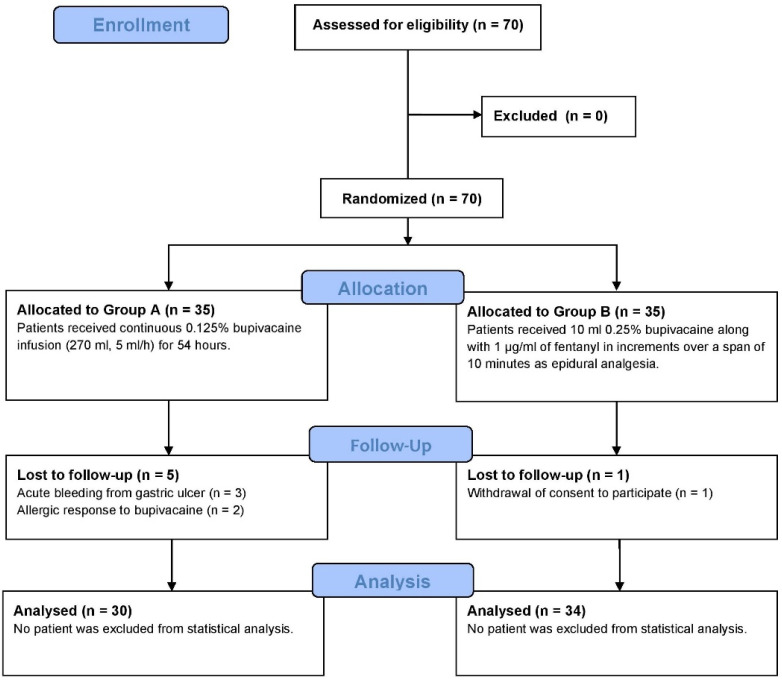
CONSORT flow diagram indicating the screening and enrolment of patients in the present clinical trial for both groups.

We systematically screened all adult patients (≥18 years) scheduled for elective open gastrectomy in our department due to malignancy or complicated ulcer disease. Eligibility criteria included patients classified as American Society of Anesthesiologists (ASA) Grades I to III. Exclusion criteria encompassed those requiring acute gastric surgery, individuals with known bupivacaine allergies, and patients who declined analgesia via RSB catheters and continuous bupivacaine infusions. Furthermore, individuals with a history of uncontrolled coagulopathy, prior laparotomy, or wound dehiscence were also excluded. Based on these criteria, a total of 70 patients qualified for inclusion in the trial (Figure 1).

### 2.2. Group Randomization

Random numbers between 1 and 70 were generated using SPSS Software v.26.0 (IBM SPSS, Inc., Armonk, NY, USA). These numbers were then ranked, with the 35 lowest assigned to Group A (receiving an RSB with continuous bupivacaine infusion) and the remaining assigned to Group B (receiving thoracic epidural analgesia). The group allocation was handled by a researcher not involved in the surgical procedures to maintain blinding. The surgeon performing the RSB received a sealed envelope containing the group assignment upon the patient’s inclusion. The anesthesiologist accessed this envelope only when the patient was brought to the operating room, determining whether an epidural catheter needed to be inserted. Additionally, investigators conducting post-operative follow-ups remained unaware of group assignments, ensuring a double-blind study design.

### 2.3. Surgical Procedure

The surgical procedure involved an upper middle laparotomy. Prior to the surgery, standard preoperative monitoring was conducted, including venous access placement, pulse oximetry, electrocardiography, and automated non-invasive arterial blood pressure measurement. General anesthesia was administered through both intravenous and endotracheal methods following standard protocols. For Group A, a bilateral rectus sheath block (RSB) was performed towards the end of the surgery, after completing the intra-abdominal stage (total gastrectomy or gastric resection with Roux-en-Y reconstruction) under the direct supervision of the surgeon. Bilateral retromuscular catheters were then placed and two ‘*Easy Pumps*’ (270 mL, 5 mL/h) containing 0.125% bupivacaine solutions were attached to the catheters by an individual who was aware of the patient’s group assignment. Patients in Group A were administered up to 288 mg of bupivacaine daily, with a maximum allowable daily dose of 400 mg [9].

For Group B, an experienced anesthetist, who was not involved in the study, performed the insertion of the epidural catheter before the initiation of general anesthesia. The procedure followed standard aseptic techniques, using an 18-gauge, 80 mm Tuohy needle from a conventional epidural set (Perifix^®^, Braum, Germany), with a midline approach. The interspace between the T9–T10 vertebrae was selected for the injection after infiltrating the skin with 2 mL of 2% lidocaine. Once the epidural space was identified using the loss of resistance method, the catheter was advanced 3 cm into the space. A test dose of 3 mL of 2% lidocaine combined with 1:200,000 adrenaline was then administered to confirm catheter placement and avoid subarachnoid puncture or misplacement into a blood vessel. If placed correctly, then 10 mL of 0.25% bupivacaine, along with 1 μg/mL of fentanyl, was injected in increments over a period of 10 min to activate the catheter. In Group B, patients received a continuous epidural infusion of 0.25% bupivacaine at a rate of 5 mL/h. Thus, both groups are comparable.

After the surgery, all patients from both groups were monitored in the intermediate care unit, with all vital signs being continuously observed.

### 2.4. Postoperative Pain Assessement

Postoperative pain levels were monitored using the Visual Analog Scale (VAS) ranging from 0 to 100 mm [10]. Postoperatively the on-duty nurses, at different time points, independently recorded pain scores regardless of the analgesic method, offering patients the opportunity to assess their pain levels. The nurses were not intentionally informed about the study; they were only tasked with recording the pain levels.

VAS scores were recorded at rest and during active movement at 4 h, 12 h, 24 h, and 48 h after the operation. If the VAS score exceeded 30 mm, then 30 mg of *Ketorolacum trometamolum* was administered intravenously. If the VAS score remained above 30 mm after 30 min, the patient was given 20 mg/1 mL of *Trimeperidine hydrochloridum* intramuscularly. The doses and frequency of pain killers and rescue analgesia provided were also recorded for both groups.

### 2.5. Postoperative Recovery Assessment

Postoperative follow-up was carried out by two investigators who were unaware of the grouping and who received sufficient training before the study. Upon returning to the ward, patients were initially allowed to drink water. On the second postoperative day, they were permitted to consume liquid foods, and by the third day, they were gradually transitioned to solid foods. Out-of-bed activities were encouraged starting on the first postoperative day to promote early mobilization. Moving from a lying position to a sitting position was defined as active movement. A physical therapist evaluated the patients on the first postoperative day, providing guidance on early rehabilitation. On the second postoperative day, patients began engaging in physical activities as recommended by the rehabilitation specialist to support patient recovery and improve mobility.

### 2.6. Data Collection

The patient’s demographic information, health status as assessed by the ASA grading, duration of surgery, and the type, frequency, and dosage of anesthesia administered during the procedure were recorded. The primary endpoints of the study were assessed immediately after surgery. Secondary outcomes were documented following the patients’ discharge from the hospital. Postoperative complications, categorized according to the Clavien–Dindo classification, were carefully monitored and recorded until the patients were discharged.

### 2.7. Statistical Analysis

Statistical analysis was performed with SPSS software version 29.0 (IBM SPSS, Armonk, NY, USA). Normality for continuous data was assessed using Shapiro–Wilk test and visually using the Q-Q plots. Continuous variable data was expressed as median and interquartile range (IQR Q1 to Q3) due to violation of normality assumption. Mann–Whitney U test was used for two independent groups or Friedman’s ANOVA with post hoc tests and Bonferroni correction for three related groups. We used Kendall’s W as effect size to quantify the degree of consistency in pain score rankings over time within each group. Values range from 0 to 1, with higher values indicating stronger temporal trends. This aids clinical interpretation by contextualizing the magnitude of within-group changes across postoperative time points.

Categorical data were analyzed using the Chi-square (χ^2^) test or Fisher’s exact test in case of violation of assumptions. A *p* value < 0.05 was considered as statistically significant. Mixed-effects modeling was performed to assess the interactions between group and time variables for continuous primary outcomes. These models were adjusted for covariates and multiple corrections.

### 2.8. Post Hoc Power Analysis

At our center we perform about 50 open gastrectomies annually, irrespective of the indications. Given the limited eligible population size, our study did not include an a priori sample size estimation. Nonetheless, we conducted a post hoc power analysis based on key outcomes using G*Power v3.1.9.7. For the primary continuous outcome of VAS score at 24 h, Group A had a mean (SD) score of 23.3 mm (11.8), while Group B had a mean (SD) score of 46.0 mm (7.0). The effect size (Cohen’s d) was calculated as 2.33, indicating a very large between-group difference. At a significance level of α = 0.05, the calculated statistical power exceeded 99.9%, confirming that the study was sufficiently powered to detect the observed difference in postoperative pain scores. Similar results were obtained for VAS scores at 48 h. Regarding the use of opioid Trimperidine hydrochloridum (binary outcome) at 24 h postoperatively, in Group A, 3.3% of patients required the opioid compared to 32.4% in Group B. At α = 0.05, the calculated statistical power was 88.9%, indicating sufficient sensitivity to detect the observed between-group difference. At 48 h, however, in Group A no patients required rescue opioid analgesia, compared to 14.7% in Group B. Despite this numerical difference, the computed power was only 38.3%, indicating that the study was underpowered at this time point to detect a difference in this magnitude.

## 3. Results

In the present study, we included a total of 64 eligible patients after accounting for patients lost to follow-up—Group A with RSB (30 patients) and Group B with epidural (34 patients). There were no significant differences observed in the distribution of age, height, weight, or BMI across the two groups (Table 1). Although, in the overall cohort we had a male predominance (39 males vs. 25 females), group-wise distribution showed no significant difference (*p* = 0.242). In terms of surgical characteristics, the epidural group had significantly longer surgical time compared to the RSB group (*p* = 0.002). Similarly, a marginally significant result was obtained in the distribution of patients based on ASA Grade. A post hoc column proportion z-test with Bonferroni correction revealed significant differences in proportion of ASA Grade I patients between Group A and Group B (37% vs. 12%, respectively; *p* < 0.05).

### 3.1. Postoperative Pain Assessment

Patients in both groups reported a gradual reduction in pain scores after surgery. Post hoc tests with Bonferroni correction following Friedman’s ANOVA showed that in Group A (RSB), there were no significant differences in pain scores between 4 h and 12 h post-surgery (*p* = 0.189). However, pain scores were significantly lower at 24 h (*p* < 0.001) and 48 h (*p* < 0.001) compared to 4 h post-surgery. This overall pattern was supported by a large effect size (Kendall’s W = 0.755), indicating strong concordance across time points.

In Group B (epidural), a significant Friedman’s ANOVA was also observed (*p* < 0.001), though post hoc comparisons with Bonferroni correction did not reveal significant pairwise differences at 12 h (*p* = 1.000) or 24 h (*p* = 0.446) compared to 4 h post-surgery. Only at 48 h did the post hoc tests show a significant reduction in pain scores compared to 4 h (*p* < 0.001). This was reflected by a small effect size (Kendall’s W = 0.285). Furthermore, cross-sectional comparisons between groups at each time point revealed that Group A consistently reported a lower VAS score than Group B (Table 2). Taken together, these findings suggest that RSB provided a more consistent and clinically meaningful reduction in postoperative pain, while epidural analgesia showed a less pronounced effect over time.

**Figure 2:**
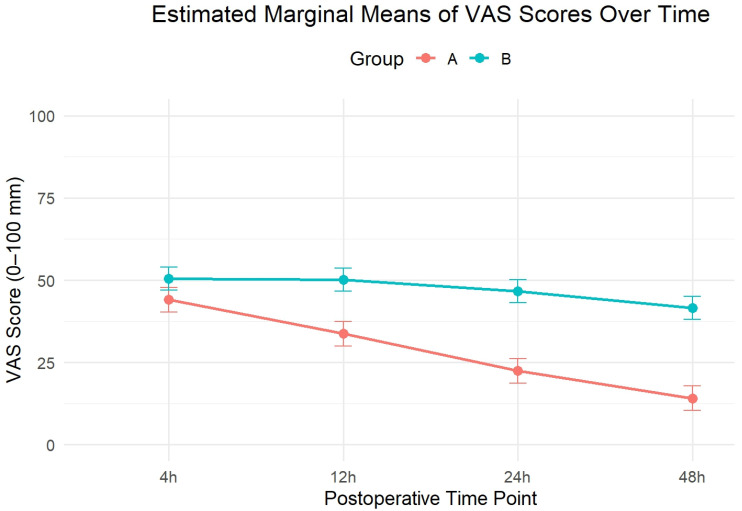
Estimated marginal means (EMMs) of postoperative pain scores measured using the VAS ID#: 87 at 4, 12, 24, and 48 h following surgery in Group A (RSB) and Group B (TEA). The analysis was performed using a linear mixed-effects model with fixed effects for group, time, and their interaction, and random intercepts for subjects. The model was adjusted for ASA classification and duration of surgery as covariates. Error bars represent 95% confidence intervals.

Next, we conducted a linear mixed-effects model to assess changes in the VAS scores over time, accounting for both within-participant repeated measures and adjusting for ASA grade and surgery duration as covariates. The analysis revealed significant main effects of the group assigned (F(1,59) = 83.9, *p* < 0.001) and time period (F(3,186) = 73.6, *p* < 0.001), as well as a significant group × time interaction (F(3,186) = 22.6, *p* < 0.001), indicating that the pain trajectories varied significantly between groups as time passed. Post hoc pairwise comparisons with Holm correction for multiple testing showed that Group A patients consistently reported significantly lower VAS scores than Group B patients at each post-operative time point: 4 h (*p* = 0.018), 12 h, 24 h, and 48 h (*p* < 0.0001; Figure 2). ASA status and surgery duration were ruled out as significant covariates in the model (*p* = 0.285 and 0.342).

### 3.2. Rescue Pain Management and Analgesia

Rescue pain management was required in both groups at all observed time points. In the RSB group, the number of Ketorolac injections administered ranged from one to three per patient, whereas in the epidural group only one injection was required per patient. Despite this, the number of patients requesting injections was consistently and significantly lower in Group A compared to Group B (Table 3). Even at 48 h post-surgery, 94% of patients in the epidural group requested rescue medication, compared to just 17% in the RSB group.

Similar observations were recorded for Trimperdine hydrochloridum injections. In the RSB group, the number of injections administered ranged from one to three per patient, whereas in the epidural group, the number of injections required ranged from one to nine per patient. The number of patients requesting injections was, however, consistently and significantly lower in Group A compared to Group B (Table 4). At the end of the observation period, none of the patients in Group A required injections while 15% of the patients in Group B requested injections.

### 3.3. Postoperative Recovery

We observed a significantly faster postoperative recovery in patients receiving RSB in comparison to those receiving TEA (Table 5). The number of post-operative hospitalization days was also significantly lower in the RSB group than the TEA group.

### 3.4. Complications

In Group B, 4 (11.7%) patients experienced severe headaches. No non-surgical or medication-related side effects were observed in Group A.

## 4. Discussion

Open gastrectomy is a major surgical procedure often associated with significant postoperative pain [11]. Effective pain management is crucial for promoting a faster patient recovery. As outlined in enhanced recovery protocols for gastrointestinal surgery, the use of multimodal, opioid-sparing analgesia and early mobilization are the two key components of an optimal postoperative strategy [12].

While only a limited number of studies have compared intermittent versus continuous RSB analgesia, a distinguishing feature of our study is that catheter placement is performed under direct visual guidance by the surgeon during the procedure [13]. Our technique eliminates the need for ultrasound guidance, which carries the risk of inaccurate catheter placement outside the intended fascial plane, potentially compromising analgesic effectiveness and postoperative outcomes. By enhancing the accuracy of catheter placement, our method may lead to improved pain control and better recovery outcomes.

Gupta et al., compared postoperative pain levels following midline laparotomies in patients undergoing intra-abdominal surgeries, evaluating both RSB and TEA [14]. The authors reported that both techniques provided comparable pain relief; however, a key distinction in the RSB group was the use of ultrasound-guided catheter placement. In separate studies, Parsons et al. and Bashandy and Elkholy, compared the RSB to opioid-based analgesia, confirming the efficacy of RSB and reporting significantly lower levels of postoperative pain in the RSB group [14,15].

In another study by Yassin and colleagues, it was reported that patients in the TEA group experienced more pronounced pain and required significantly higher opioid supplementation compared to those in the RSB group receiving intermittent bupivacaine administration [16]. This outcome may be attributed to less accurate catheter placement under ultrasound guidance and the use of intermittent, rather than continuous, bupivacaine infusion. Notably, the same study also found that patients in the RSB group had a considerably faster postoperative recovery, and a shorter hospital stay compared to the TEA group. In the study by Gupta et al., which references the findings of Yassin and colleagues, these differences are discussed in the context of dosage variation and the recommendation to use ropivacaine, owing to its longer duration of action and lower toxicity profile.

Epidural anesthesia is known to be associated with a range of complications, some of which can be severe. Reported issues include neurological symptoms (12.9%), abnormalities at the epidural insertion site (5.6%), complete catheter migration (4.4%), the need for epidural catheter replacement (3.3%), and inadvertent dural puncture (1.7%) [17]. In our study, four patients (11.7%) in the TEA group experienced severe headaches; however, no other serious neurological complications were observed.

In the current clinical landscape, where patients are frequently prescribed antiplatelet or anticoagulant medications for cardiovascular disease prevention, TEA also poses a significantly increased risk of complications such as bleeding and hematoma formation. In such cases, RSB analgesia has been recommended as the preferred alternative due to its safer profile [12]. In our study, all patients were scheduled for elective surgery, and no cases of bleeding or hematoma were observed. It is important to note that many TEA-related complications are closely linked to the anesthesiologist’s level of experience and technical skill. In our cohort, all TEA procedures were performed by highly experienced anesthesiologists who regularly manage patients undergoing complex abdominal and oncological surgeries.

A significant factor included in our analysis, alongside the duration of the surgical procedure, was the time from the initiation of epidural anesthesia placement. When comparing Group A and Group B, the total operative time was, on average, approximately 35 min longer in Group B. This increase is primarily attributed to the time required for epidural catheter insertion and the subsequent pause before surgery could begin. In contrast, pain management using a continuous retromuscular Bupivacaine infusion typically reduced operative time by the time it took to insert and place TEA. Nonetheless, one potential complication of the RSB technique is catheter fixation with sutures, which may necessitate reoperation, wound revision, or catheter removal [17]. However, no such complications were observed in the RSB group in our study.

The most notable benefit, also reported in similar studies, is the significantly earlier patient recovery observed in the RSB group compared to the TEA group [16]. In our study, the average postoperative hospital stay was six days for the RSB group, compared to nine days for the TEA group. Early recovery in the RSB group was further reflected in earlier return of bowel function, with both the first passage of flatus and first bowel movement occurring, on average, two days earlier than in the TEA group. Recovery outcomes in the TEA group were comparable to those typically seen with opioid-based analgesia, as described by another study conducted at our tertiary care center [1].

Despite our promising results, our study leaves room for further refinement. Pain assessment was performed using the VAS. However, future studies could benefit from employing objective tools such as the Analgesia Nociception Index (ANI) device, which offers a more standardized measurement of pain response. Additionally, tailoring the bupivacaine dosage based on plasma concentration could potentially affect pain control outcomes. A broader multicenter study would be essential, involving not only university hospitals but also regional hospitals, where anesthesiologists with less experience sometimes work. This could potentially lead to a higher risk of complications, including severe ones, especially with epidural anesthesia, which would serve as further evidence that surgical placement of the RSB catheter is both straightforward and precise, with minimal risk of complications. Future investigations should also explore the comparative efficacy of bupivacaine and ropivacaine, the latter of which has been shown in several studies to provide a longer duration of analgesia and a more favorable toxicity profile.

## 5. Conclusions

Both TEA and bilateral RSB with bupivacaine infusion are effective and reliable methods of postoperative analgesia that support early patient recovery. However, RSB offers several advantages, including faster recovery, reduced opioid consumption, and decreased need for rescue analgesia. Additionally, it is associated with shorter hospital stays, resulting in cost savings and a lower risk of complications. Given the potential for serious complications with TEA and the requirement for skilled anesthesiologists, RSB could represent a safer and more practical option, offering comparable analgesic efficacy with clear benefits in early rehabilitation and recovery.

## Figures and Tables

**Table 1:** Patient and surgical characteristics in both groups.

Characteristics *	Group A (*n* = 30)	Group B (*n* = 34)	*p* Value
**Sex**			
Male	16 (53%)	23 (68%)	0.242 ^‡^
Female	14 (47%)	11 (32%)	
**Age (years)**	71 (63 to 83)	71 (64 to 75)	0.427 ^†^
**Height (cm)**	176 (165 to 182)	179 (172 to 185)	0.055 ^†^
**Weight (kg)**	76 (60 to 88)	78 (70 to 86)	0.423 ^†^
**Body mass index (kg/m^2^)**	23.9 (21 to 29)	23.7 (21 to 30)	0.696 ^†^
**ASA Grade**			
Grade I	11 (37%)	4 (12%)	0.049 ^‡^
Grade II	14 (47%)	19 (56%)	
Grade III	5 (16%)	11 (32%)	
Duration of surgery (mins)	180 (160 to 205)	215 (175 to 310)	0.002 ^†^

* Numerical values are presented as median (Q1 to Q3). Categorical values are presented as *n* (%). ^†^ Derived from Mann–Whitney U test. ^‡^ Derived from Pearson Chi-square (χ^2^) test.

**Table 2:** Post-operative pain scores measured using visual analog scale (VAS; 0–100 mm).

Time After Surgery *	Group A (*n* = 30)	Group B (*n* = 34)	*p* Value ^†^
0–4 h	40 (30 to 60)	50 (40 to 53)	0.079
4–12 h	40 (20 to 40)	50 (40 to 53)	<0.001
12–24 h	20 (18 to 30)	45 (40 to 50)	<0.001
24–48 h	10 (10 to 20)	40 (40 to 40)	<0.001
***p* value ^‡^**	<0.001	<0.001	-

* Numerical values are presented as median (Q1 to Q3). ^†^ Derived from Mann–Whitney U test. ^‡^ Derived from Friedman’s ANOVA.

**Table 3:** Requirement of *Ketorolacum trometamolum* injections in both groups.

Injections Given *	Group A (*n* = 30)	Group B (*n* = 34)	*p* Value
**0–4 h**			
Yes	26 (87%)	34 (100%)	0.043 ^‡^
No	4 (13%)	0 (0%)	
**4–12 h**			
Yes	19 (63%)	34 (100%)	<0.001 ^†^
No	11 (37%)	0 (0%)	
**12–24 h**			
Yes	10 (33%)	33 (97%)	<0.001 ^†^
No	20 (67%)	1 (3%)	
**24–48 h**			
Yes	5 (17%)	32 (94%)	<0.001 ^†^
No	25 (83%)	2 (6%)	

* Values are presented as *n* (%). ^†^ Derived from Pearson Chi-square (χ^2^) test. ^‡^ Derived from Fisher’s Exact test due to violation of assumptions.

**Table 4:** Requirement of *Trimperidine hydrochloridum* injections in both groups.

Injections Given *	Group A (*n* = 30)	Group B (*n* = 34)	*p* Value
**0–4 h**			
Yes	8 (27%)	16 (47%)	0.093 ^†^
No	22 (73%)	18 (53%)	
**4–12 h**			
Yes	4 (13%)	19 (56%)	<0.001 ^†^
No	26 (87%)	15 (44%)	
**12–24 h**			
Yes	1 (3%)	11 (32%)	0.003 ^†^
No	29 (97%)	23 (68%)	
**24–48 h**			
Yes	0 (0%)	5 (15%)	0.055 ^‡^
No	30 (100%)	29 (85%)	

* Values are presented as *n* (%). ^†^ Derived from Pearson Chi-square (χ^2^) test. ^‡^ Derived from Fisher’s Exact test due to violation of assumptions.

**Table 5:** Assessment of post-operative recovery measures.

Characteristic *	Group A (*n* = 30)	Group B (*n* = 34)	*p* Value ^†^
First gas (days)	2 (2 to 3)	5 (4 to 5)	<0.001
First stool (days)	3 (3 to 4)	5 (5 to 6)	<0.001
First out-of-bed activity (days)	3 (2 to 3)	4 (4 to 5)	<0.001
Post-operative days	6 (5 to 7)	9 (7 to 11)	<0.001

* Numerical values are presented as median (Q1 to Q3). ^†^ Derived from Mann–Whitney U test.

## Data Availability

The data are available from the corresponding author on reasonable request.
