# Correction: Opincans et al. Comparison of Bilateral Rectus Sheath Block and Thoracic Epidural Analgesia for Postoperative Pain Control After Open Gastrectomy: A Randomized Controlled Trial. *Medicina* 2025, *61*, 1695

**DOI:** 10.3390/medicina62091729

**Published:** 2026-09-08

**Authors:** Janis Opincans, Igors Ivanovs, Aleksejs Miscuks, Janis Pavulans, Elina Zemite, Agris Rudzats, Zurabs Kecbaja, Aleksejs Kaminskis

**Affiliations:** 1 Department of Surgery, Riga East Clinical University Hospital, 1038 Riga, Latvia; 2 Faculty of Medicine, University of Latvia, 1004 Riga, Latvia; 3 Faculty of Medicine, Riga Stradins University, 1007 Riga, Latvia

In the original publication [1], the authors incorrectly cited clinical trial registration details that belonged to another study [2], and the ethics approval code and the corresponding date were incorrect. The authors failed to register prospectively on ClinicalTrials.gov because of an administrative oversight. These have been corrected to reflect the correct retrospective registration details and the accurate ethics approval information.

## 2. Materials and Methods

Between October 2021 and December 2024, we planned and undertook a randomized controlled trial at Riga East Clinical University Hospital (RAKUS) in Riga, Latvia. The study protocol was reviewed and approved by the Research Ethics Committee of the University of Latvia (decision no. 02022021, dated 2 February 2021), and was conducted with the approval of the Riga East University Hospital’s scientific department (approval no. AP-21/21, dated 25 January 2021). In accordance with the Declaration of Helsinki (2013), all participants provided written informed consent before taking part in the study. The trial protocol was retrospectively registered in the ClinicalTrials.gov database (ID: NCT07634731), as prospective registration did not occur because of the authors’ administrative oversight.

**Institutional Review Board Statement:** The study followed the guidelines described in the Helsinki Declaration, 2013. The study protocol was reviewed and approved by the Research Ethics Committee of the University of Latvia (decision no. 02022021, dated 2 February 2021), and was conducted with the approval of the Riga East University Hospital’s scientific department (approval no. AP-21/21, dated 25 January 2021).

The authors state that the scientific conclusions are unaffected. This correction was approved by the Academic Editor. The original publication has also been updated.
